# Limitations in assessing nerve growth factor levels in aqueous humor samples from human eyes

**DOI:** 10.1186/1756-0500-1-22

**Published:** 2008-06-05

**Authors:** Kakarla V Chalam, Rajesh K Sharma, Ravi K Murthy

**Affiliations:** 1 Department of Ophthalmology, University of Florida College of Medicine, Jacksonville, Florida

## Abstract

**Background:**

Nerve growth factor (NGF) helps in the healing and survival of ganglion cells, photoreceptors, and optic nerve after injury and has been implicated to have a role in pathophysiology of glaucoma. So far, in animal studies, injury to iris in vitro has revealed an increase in NGF levels in aqueous. There is a great interest in investigating the levels of NGF in human aqueous in glaucomatous eyes, as suggested by animal studies, to gain a better understanding of the pathophysiology of glaucoma.

**Findings:**

In this study, we examined the presence of NGF levels in aqueous humor collected from human eyes and the limitations in determining the NGF levels in human samples. NGF was assessed by ELISA immunoassay in undiluted aqueous samples collected from 32 consecutive patients undergoing surgery for cataract (control) or primary open angle glaucoma (POAG). Recombinant NGF was used as positive control. NGF levels were below undetectable levels in aqueous humor from eyes with POAG and controls by immunoassay. Less than 10% of samples had detectable NGF levels and these were considered outliers.

**Conclusion:**

Our result highlights the undetectable levels of NGF in human aqueous samples.

## Introduction

Aqueous occupies the anterior and posterior chambers of the eye and nourishes the lens and the corneal endothelium. The aqueous composition resembles that of plasma, but contains much less proteins and glucose. It has higher levels of lactic and ascorbic acids. A number of studies have implicated nerve growth factor (NGF) in pathophysiology of glaucoma [1-3]. NGF receptors have been demonstrated on human trabecular meshwork [2,4] make it interesting to investigate changes in NGF levels in aqueous samples from patients. In this study we evaluated the feasibility of measuring NGF levels in aqueous samples using a commonly used ELISA kit.

## Methods

The study was approved by the Institutional Review Board, University of Florida, Jacksonville. An informed consent was obtained from all the patients included in the study. Aqueous samples were collected from 32 consecutive patients undergoing surgery for cataract (control) or primary open angle glaucoma (POAG). At the time of surgery, after preparing the eye, undiluted aqueous sample was collected from the anterior chamber by a 27 gauge insulin syringe. Samples were immediately frozen at -80°C and thawed immediately before assay. 18 patients had POAG and 14 patients had cataract.

Most frequent sample size that could practically be collected (mode) was 50 μL of aqueous. ELISA immunoassay was performed to quantify the NGF levels in the aqueous (Promega, Madison, WI). In this kit, flat-bottom 96-well plates are coated with Anti-NGF Polyclonal Antibody which binds soluble NGF. The plates are covered and incubated overnight at 4°C. The following day the plates are washed and blocking buffer added in the wells. Human beta-NGF (PeproTech, Rocky Hill, NJ) was used as external positive control (in addition to NGF provided by the kit for standard curve) and buffer as negative control. Due to limitation of the sample size, 50 μL of undiluted samples were used to run the assay. All samples could be run in single, except the standards and controls. Most samples were in the range of 50 μL, therefore this quantity was used as a standard sample size. Some samples had smaller quantity (40 μL) where this sample was diluted to make 50 μl and eventually dilution factor was accounted for in the final calculations. Following six hours of incubation with shaking at room temperature, the plate was washed and secondary antibody (anti NGF mAb) was added. Plate was incubated overnight without shaking at 4°C. Next day, plate was washed and Anti-Rat IgG, HRP conjugate was added. Following 2.5 hours of incubation at room temperature and wash, TMB solution was added. Reaction was stopped with 1N hydrochloric acid and the plate read at A450 within 30 minutes.

## Results

In the POAG samples (n = 18) the mean age of the subjects was 74.2 yrs and in controls (n = 14) the mean age of the subjects was 69 years.

**Figure 1:**
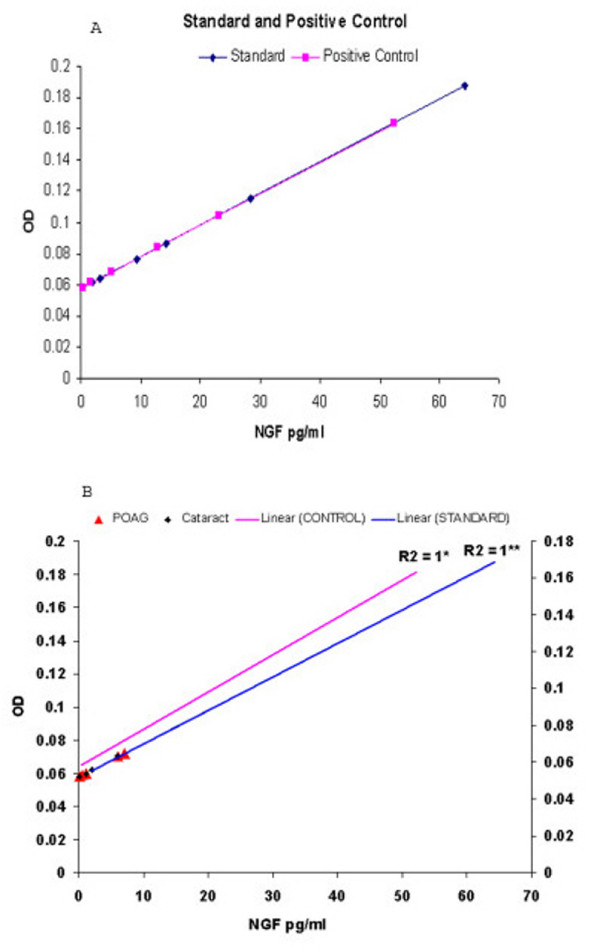
A) Standard curve as well as external positive control values were linear below the 10 pg/ml NGF levels, comparable to 7.8 pg/ml sensitivity level of the Elisa kit. B) ELISA on the aqueous samples from patients' undergone cataract and POAG surgery. Standard curve has a R^2 ^value of 1 and the recombinant NGF kept as positive control show predicted values (R^2 ^= 1). In both control (green diamond) and POAG (red triangles) samples NGF was below the sensitivity of the kit.

With undiluted samples, the typical average levels of NGF were 0.91 pg/ml (standard error 0.52) in aqueous samples of patients with POAG and 0.82 pg/ml (standard error 0.47) in aqueous samples of controls. Levels were considered undetectable as these were below the detection sensitivity of the kit (7.8 pg/ml). In each experiment external recombinant NGF controls were kept and their values (coefficient of determination; R^2 ^= 1) were detected as predicted (Fig. 1). However, in both glaucoma and control groups approximately 5.5% and 7.1% of samples had NGF values as high as 75.3 pg/ml and 17.9 pg/ml respectively.

Even in pilot experiments quantities ranging from 15 to 50 μl and dilutions ranging from 1:1.25–3.3 were used but NGF levels were found to be undetectable and on rare occasions when larger quantities were available (up to 100 μL; n = 2) the NGF levels were still undetectable.

## Discussion

Results show that the NGF was present in aqueous humor in a concentration below 1 pg/ml. These levels are below the sensitivity limit of the kit used, thus they were considered undetectable. However, since the standard curve as well as external positive control values were linear below the 7.8 pg/ml sensitivity levels, it is possible that the detected levels represent actual NGF values. The results from aqueous samples are considered reliable because the positive controls give predictable values. Quantity of sample size available from patients posed a problem; however, even when 100 μL of aqueous was available the NGF levels were undetectable. The same experiment was performed two more times with additional samples and the results were comparable. This is consistent with low quantities of proteins present in aqueous [5].

Several methods have been described in the literature to enhance detectability of a protein [6]. Even in the presence of low levels of starting proteins, sample dilutions, possibly by diluting other interfering proteins, can some times improve detectability. However, even with dilution in pilot studies NGF levels in aqueous remained below sensitivity levels. Methods have been described to extract NGF in samples by acidification. This method requires acid treatment and readjustment of the pH [7]. Pretreatment or pH shock extraction was not feasible because of the small sample size. Other possibility is to concentrate the sample with freeze-dry method. This method could only be used if we pooled the samples from different patients. This will however preclude our aim of detecting NGF levels in individual subjects.

The samples with higher values were considered outliers (with respect to patient groups of control and POAG) because (1) these values were rare and highly inconsistent with others in the group (2) they were found with equal frequency in control and glaucomatous eyes. Histories of outliers were reviewed to identify any apparent cause but none was identified.

Our results highlight the difficulties in assessing NGF levels in aqueous. An alternate strategy could be to pool the samples from different patients (but from same group) in order to increase the sample volume and therefore the detectability. However, results also show that outliers may skew the results obtained by pooling the samples. The outliers may be a result of variables at the time of collection of sample (e.g. per operative trauma, bleeding, iris handling etc). There are reports in the literature where NGF levels have been assessed in animals [3,8] and in humans [1].

Our result highlights the limitations in collection of 100 μl of human aqueous sample for Elisa assay, to determine the nerve growth factor levels. This result differs from limited reports where NGF levels have been reported in aqueous samples [1,3,8]. However, in these reports methodological details are not clear if 100 microlitres of aqueous was obtained by pooling the samples. Considering these limitations, other technologies such as multiplex assays might be more useful.

## Competing interests

The authors declare that they have no competing interests.

## Authors' contributions

KVC conceptualized the design of the study, revised the manuscript and verified its intellectual content. RKS was involved in conducting the experiments and drafting of the manuscript. RKM was involved in drafting and revision of the manuscript. All authors read and approved the final manuscript.
